# A novel ROS-Related chemiluminescent semiconducting polymer nanoplatform for acute pancreatitis early diagnosis and severity assessment

**DOI:** 10.1186/s12951-023-01937-9

**Published:** 2023-05-31

**Authors:** Yuhang Li, Baoli Yin, Yinghui Song, Kang Chen, Xu Chen, Yujing Zhang, Nanhui Yu, Chuang Peng, XiaoBing Zhang, Guosheng Song, Sulai Liu

**Affiliations:** 1 Department of Hepatobiliary Surgery, The First Affiliated Hospital of Hunan Normal University, 61 Jiefang Road, Changsha, 410005 Hunan China; 2 Central Laboratory, Hunan Provincial People’s Hospital (The First Affiliated Hospital of Hunan Normal University), Changsha, 410015 China; 3 State Key Laboratory of Chemo/Bio-Sensing and Chemometrics, College of Chemistry and Chemical Engineering, Hunan University, Changsha, 410082 China; 4 Key Laboratory of Molecular Epidemiology of Hunan Province, School of Medicine, Hunan Normal University, Changsha, China; 5 Department of Gastrointestinal Surgery, the Second Xiangya Hospital, Central South University, Changsha, China

**Keywords:** Semiconducting polymer nanoparticles, Chemiluminescence imaging, Acute pancreatitis, Early diagnosis, Severity assessment

## Abstract

Acute pancreatitis (AP) is a common and potentially life-threatening inflammatory disease of the pancreas. Reactive oxygen species (ROS) play a key role in the occurrence and development of AP. With increasing ROS levels, the degree of oxidative stress and the severity of AP increase. However, diagnosing AP still has many drawbacks, including difficulties with early diagnosis and undesirable sensitivity and accuracy. Herein, we synthesized a semiconducting polymer nanoplatform (SPN) that can emit ROS-correlated chemiluminescence (CL) signals. The CL intensity increased in solution after optimization of the SPN. The biosafety of the SPN was verified in vitro and in vivo. The mechanism and sensitivity of the SPN for AP early diagnosis and severity assessment were evaluated in three groups of mice using CL intensity, serum marker evaluations and hematoxylin and eosin staining assessments. The synthetic SPN can be sensitively combined with different concentrations of ROS to produce different degrees of high-intensity CL in vitro and in vivo. Notably, the SPN shows an excellent correlation between CL intensity and AP severity. This nanoplatform represents a superior method to assess the severity of AP accurately and sensitively according to ROS related chemiluminescence signals. This research overcomes the shortcomings of AP diagnosis in clinical practice and provides a novel method for the clinical diagnosis of pancreatitis in the future.

## Introduction

Acute pancreatitis (AP) is one of the most common acute conditions affecting the abdomen [1, 2]. The pathological basis of AP is the secretion of the enzymeogen prolyl protease, trypsinogen, and amylase from pancreatic acinar cells (PACs) to the pancreatic duct [3]. These enzymes are prematurely activated in PACs and self-digest in the pancreas. According to its severity, AP is divided into mild acute pancreatitis (MAP) and severe acute pancreatitis (SAP) [4]. Approximately 20% of AP progresses to SAP in a short period of time, and is accompanied by pancreatic tissue injury and necrosis [5, 6]. Moreover, SAP eventually leads to multiorgan failure in humans, mainly affecting the lungs, kidneys and liver [7]. Studies have shown that dysfunction of the above organs accounts for approximately 40% of deaths caused by SAP and its complications [6, 8]. In recent decades, the methods of diagnosing AP have changed significantly. In addition to computed tomography (CT) and magnetic resonance imaging (MRI), endoscopic diagnosis, minimally invasive treatment and multidisciplinary diagnoses have been developed [9, 10]. However, AP diagnosis still relies mainly on laboratory tests and imaging, which has certain drawbacks [11, 12]. For example, the limitations of using serum amylase and lipase diagnostic tests are that they are unable to distinguish between some specific types of AP (such as amylase levels that can be normal in patients with alcoholic or hypertriglyceridaemia pancreatitis) [13]; imaging tests such as CT and MRI are not sensitive to early AP [9];and a contrast- enhanced CT scan is required to diagnose necrotizing pancreatitis, and necrosis might not develop until 72 h after symptom onset [14]. For this reason, obtaining a CT scan within 72 h of symptom onset is discouraged by published guidelines, including those of the American College of Gastroenterology and American Gastroenterology Association [13, 15]. Thus, the diagnosis of AP is a challenge, especially in the early stage.

An increasing number of studies have suggested that oxidative stress plays a key role in the progression of AP [12, 13].There is a positive correlation between oxidative stress and AP [16]. Studies have shown that oxidative stress is an imbalance between the oxidative and antioxidative systems of cells and tissues. Oxidative stress is a result of the overproduction of free radicals from oxidation and associated reactive oxygen species (ROS) [15–18]. The degree of oxidative stress is positively correlated with ROS content [17]. The balance between ROS production and scavenging is maintained through a variety of mechanisms in the body and plays an important role in maintaining the stability of the body [18]. Therefore, failure to scavenge ROS leads to an oxidative stress response and disrupts the free radical production-scavenging balance, which causes oxidative damage to proteins, DNA, and lipids and further accelerates the progression of AP [14, 19, 20]. It should be noted that there is still a lack of a facile method to assess the content of ROS in real time to determine the severity of AP.

Currently, many optical methods have been developed for in vivo imaging, including fluorescence, persistent luminescence, chemiluminescence (CL) and others [21, 22]. Inconveniently, most of these methods have the drawbacks of a required external light source for excitation and self-background interference. CL imaging that relies on energy released from a chemical reaction has been promising for noninvasive detection with negligible background noise as a result of the absence of laser excitation [23–25]. However, there are still shortcomings that hamper most CL systems for in vivo imaging applications, such as extremely toxic reactions with traditional CL systems (e.g., NaNO_2_ + H_2_O_2_;KMnO_4_ + H_2_O_2_; and NaIO_4_ + H_2_O_2_) [26]; the difficulty of clearance of inorganic quantum dot CL systems by the body [27]; and the weak, short emission of luminol systems [28, 29]. Excitingly, peroxyoxalate-based chemiluminescent resonance energy transfer (CRET) systems have recently been developed to detect H_2_O_2_ associated with peritonitis, neuroinflammation, and liver injury in vivo [30, 31]. Specifically, during the CL process, bis (2,4,5-trichloro-6-(pentyloxycarbonyl) phenyl) oxalate (CPPO) as a peroxyoxalate CL substrate is rapidly decomposed by H_2_O_2_ to form the high-energy intermediate 1,2-dioxetanedione that can excite nearby fluorescent molecules to emit light through a chemically initiated electron-exchange luminescence (CIEEL) mechanism [32, 33].

In this study, we present a novel method for diagnosing early AP and assessing its severity, and further demonstrate the feasibility and effectiveness of this diagnostic method. We designed and synthesized an integrated semiconducting polymer nanoplatform (SPN) with ROS-correlated CL to assess the severity of AP. The nanoplatform was used to explore the relationship between CL intensity and ROS content in solution, further establishing an excellent correlation between CL imaging intensity and AP severity in vivo. Thus, our nanoplatform may be able to provide a new diagnostic method for AP in the clinical setting, and more effective treatment options could be proposed due to the efficiency and sensitive assessment of AP diagnosis.

## Materials and methods

All reagents and chemicals were purchased from commercial suppliers. Poly (2,7-(9,9s and bis (thiophen-2-yl) benzo-2,1,3-thiadiazole) (PFODBT), poly (styrene-co-maleic anhydride).

(PSMA) (Mw = 1600) and Pluronic F-127 were purchased from Sigma‒Aldrich. Tetrahydrofuran (THF) and bis (2,4,5-trichloro-6 (pentyloxycarbonyl)phenyl) oxalate (CPPO) were purchased from Macklin. 1,2-Dimyristoyl-sn-glycero-3-phosphoethanolamine-N-[methoxy (polyethylene glycol) (DSPE-PEG, Mw = 2000) was purchased from Shanghai Tuo Yang Biotechnology Co. Ltd. Caerulein was purchased from APEXBIO.

Transmission electron microscopy (TEM) images were obtained with a JEM-2100 F instrument (JEOL). Dynamic light scattering (DLS) was performed on a Malvern Zetasizer Nano ZS90 (Malvern). Absorbance values were recorded by ultraviolet‒visible absorption spectroscopy (UV-1800, Shimadzu) or microplate reader (SpectraMax iD3). Fluorescence spectra were recorded by fluorescence spectrophotometer (F-7000, HITACHI).

The fluorescent images were collected by a VILBER FUSION-FX7. EDGE V0.70with an exposure time of 5 s. All CL images were collected by an IVIS spectrum imaging system (Lumina XR) under bioluminescence modes with an open filter. CL images were analysed by ROI analysis using Living Image 4.0 software. The imaging parameters were as follows: bioluminescence modes open filter exposure time of 10 or 30 s, field of view of C.

All mice were purchased from Hunan SJA Laboratory Animal Co., Ltd. All animal experiments were carried out in accordance with the relevant laws and approved by the Institutional Animal Care and Use Committee of Hunan Provincial People’s Hospital (The First Affiliated Hospital of Hunan Normal University).

### Synthesis of various semiconducting polymer nanoparticles

We used nanoprecipitation to assemble hydrophobic organic molecules into nanoparticles through the hydrophobic ends of amphiphilic surfactants, and the hydrophilic ends were exposed to enhance dispersion in water.

First, a mixed solution was prepared by dissolving various masses of PFODBT, CPPO and surfactant (DSPE-PEG, F127, or PSMA) into 1 mL of THF, by sonication. Second, the above solution was rapidly injected into water (9 mL) under continuous sonication for 10 min. The THF was evaporated after 15 min of rotary evaporation. The resulting nanoparticles were washed with water three times using a 100 K centrifugal filter tube and used immediately after synthesis.

For the synthesis of CPPO@PFO nanoparticles (CPPO/PFO = 40; 0.25 mg/mL PFO), a mixed solution was prepared by dissolving 0.25 mg of PFODBT, 10 mg of CPPO, and 10 mg of DSPE-PEG in 1 mL of THF.

### CL imaging in vitro

To investigate selectivity, 50 µL of CPPO@PFO (CPPO/PFO = 40, 0.25 mg/mL of PFO) was mixed with 50 µL of different substances (1 µmol/mL) such as GSH, H_2_O_2_, ClO^−^, O_2_^·−^ and ·OH, which were prepared by directly diluting commercially available GSH, H_2_O_2_, NaOCl, KO_2_ and KOH solution with water (1 µmol/mL).

For CL imaging in solution, 50 µL of H_2_O_2_ (200 µmol/mL) was mixed with 50 µL of CPPO@PFO (CPPO/PFO = 40; 0.25 mg/mL PFO) with different surfactants (DSPE-PEG, F127, and PSMA; the concentrations of surfactants were 10 mg/mL). For CL imaging of different proportions of CPPO@PFO (CPPO/PFO = 5,10,20,40 and 60), 50 µL of CPPO@PFO (0.25 mg/mL PFO) was mixed with H_2_O_2_ (200 µmol/mL). Then, for CL imaging of different concentrations of PFODBT (0.05 mg, 0.1 mg, 0.25 mg, 0.5 mg and 1 mg), 50 µL of CPPO@PFO (CPPO/PFO = 40) was mixed with H_2_O_2_ (200 µmol/mL). Finally, 50 µL of the optimal proportion of CPPO@PFO (CPPO/PFO = 40; 0.25 mg/mL PFO),was mixed with H_2_O_2_ (25 µmol/mL, 50 µmol/mL, 100 µmol/mL, 200 µmol/mL and 400 µmol/mL). Immediately after addition, CL images were collected using an acquisition time of 10 s.

### Correlation between CPPO@PFO and cytotoxicity in vitro

Mouse-derived pancreatic exocrine cells (AR42J) were cultured in Ham’s F-12 K, medium containing 20% FBS and 1% P/S. The cells were cultured in T-25 flasks for cell inheritance at 37 °C under 5% CO_2_ in a constant temperature incubator. For CPPO@PFO experiments, AR42J cells preinoculated into 96-well plates were incubated with 190 µL of fresh Ham’s F-12 K containing CPPO@PFO (0.05 mg, 0.1 mg, 0.25 mg, 0.5 mg, or 1 mg). After coculture for approximately 24 h, the cells were examined to determine viability by the CCK-8 assay.

### Correlation between CPPO@PFO and blood compatibility in vitro

Two milliliters -of blood were drawn from healthy people and centrifuged at 2000 r/min for 5 min, the upper serum was discarded, and the blood cells were washed three times with PBS. Finally, 1 ml of red blood cell suspension was obtained. Then, 0.1 mL of red blood cells (concentration: 10^9^/ml) were cultured in different solutions (H_2_O, NaCl, DMEM, RPMI- 1640, PBS, and CPPO@PFO). After coculture for approximately 4 h, the haemolysis of the red blood cells in the different groups was observed.

### Correlation between CPPO@PFO and biosafety in vivo

Two groups (control and CPPO@PFO) of mice (ICR, female, 6–8 weeks) received treatment with NaCl or CPPO@PFO (CPPO/PFO = 40; 0.25 mg/mL PFO). After intravenous (i.v.) injections, the mouse body weights were continuously recorded for 30 days. The serum of the mice was collected and stored at -80 °C. The main organs (heart, liver, kidney, lung, and pancreas) of the mice from each group were harvested. These tissues were fixed in 4% formalin for haematoxylin and eosin (H&E) staining.

### Correlation between CL imaging and severity of acute pancreatitis in vivo

Female ICR mice received intraperitoneal injections of caerulein (50 µg/kg/h) 7 or 12 times. One hour after the last injection, three groups of mice (control, MAP and SAP groups) received CPPO@PFO treatment (CPPO/PFO = 40; 0.25 mg/mL PFO). After i.v. injection, CL images were obtained at different points in time using 30 s of acquisition. The mouse body weights were continuously recorded for 24 h. The serum of the mice was collected and stored at -80 °C. The main organs (heart, liver, kidney, lung, and pancreas) from mice from each group were harvested 30 min after treatment. These tissues were fixed in 4% formalin for H&E staining.

### Blood indicator measurements

ELISAs were conducted using commercial kits to measure blood indicators. The mouse serum collected before was used and the ELISA assay was performed according to the manufacturer’s protocol.

### Data statistics and analysis

All data in this study are expressed as the mean ± standard deviation (SD). GRAPHPAD software was used to compare the experimental data. p values equal to or less than 0.05 were considered statistically significant. Standard symbols are presented as *P < 0.05, **P < 0.01, ***P < 0.001, and ns, not significant.

## Results

### Synthesis and characteristics of the semiconducting polymer nanoplatform

**Fig. 1:**
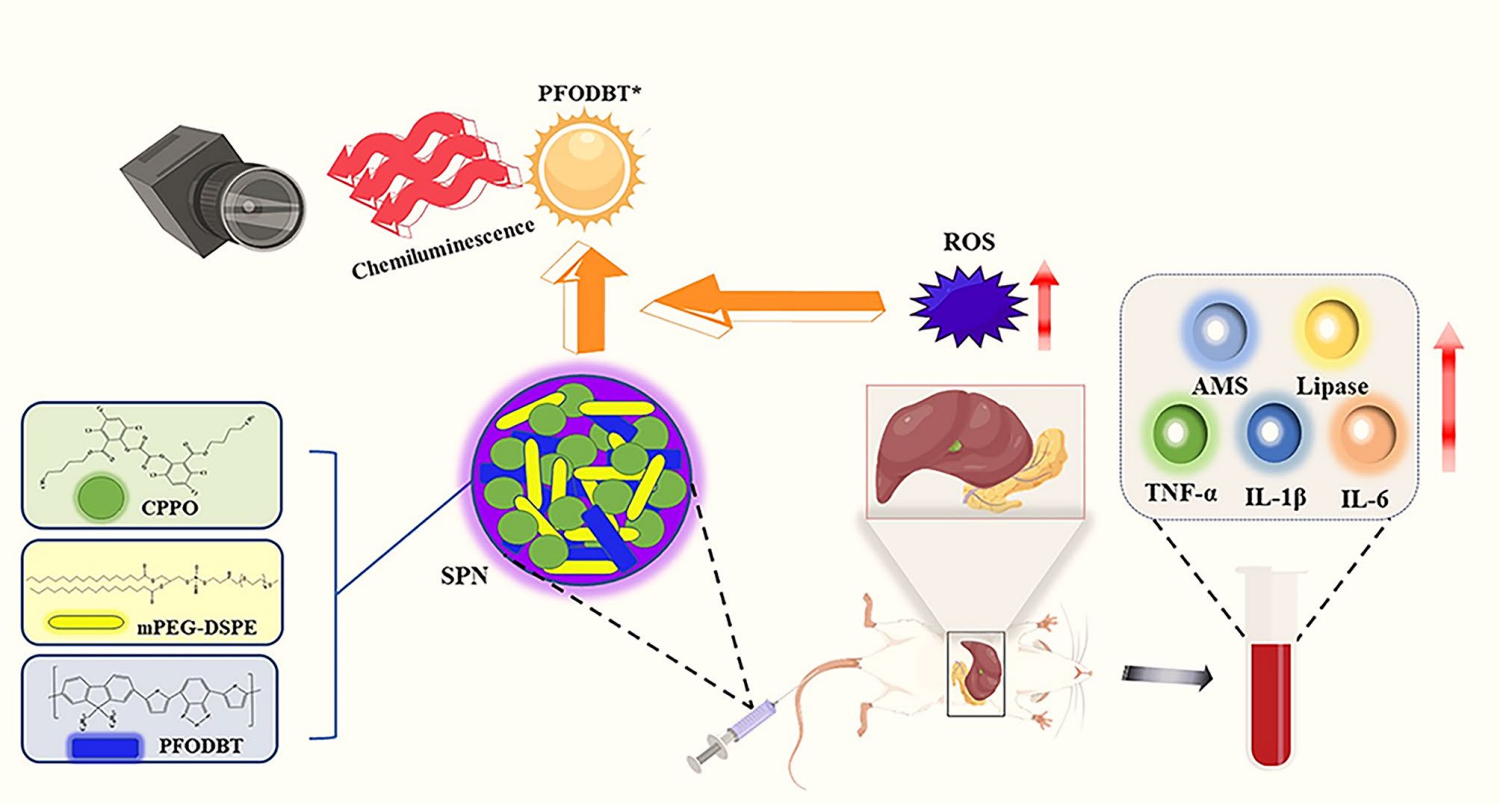
Schematic illustration of the semiconducting polymer nanoplatform to assess the severity of acute pancreatitis. This nanoplatform can establish a good relationship between the CL imaging intensity and the content of ROS and thereby an excellent correlation between the CL intensity and the degree of oxidative stress in vivo. The nanoplatform can be used for the early diagnosis of AP and to assess the severity of AP.

**Fig. 2:**
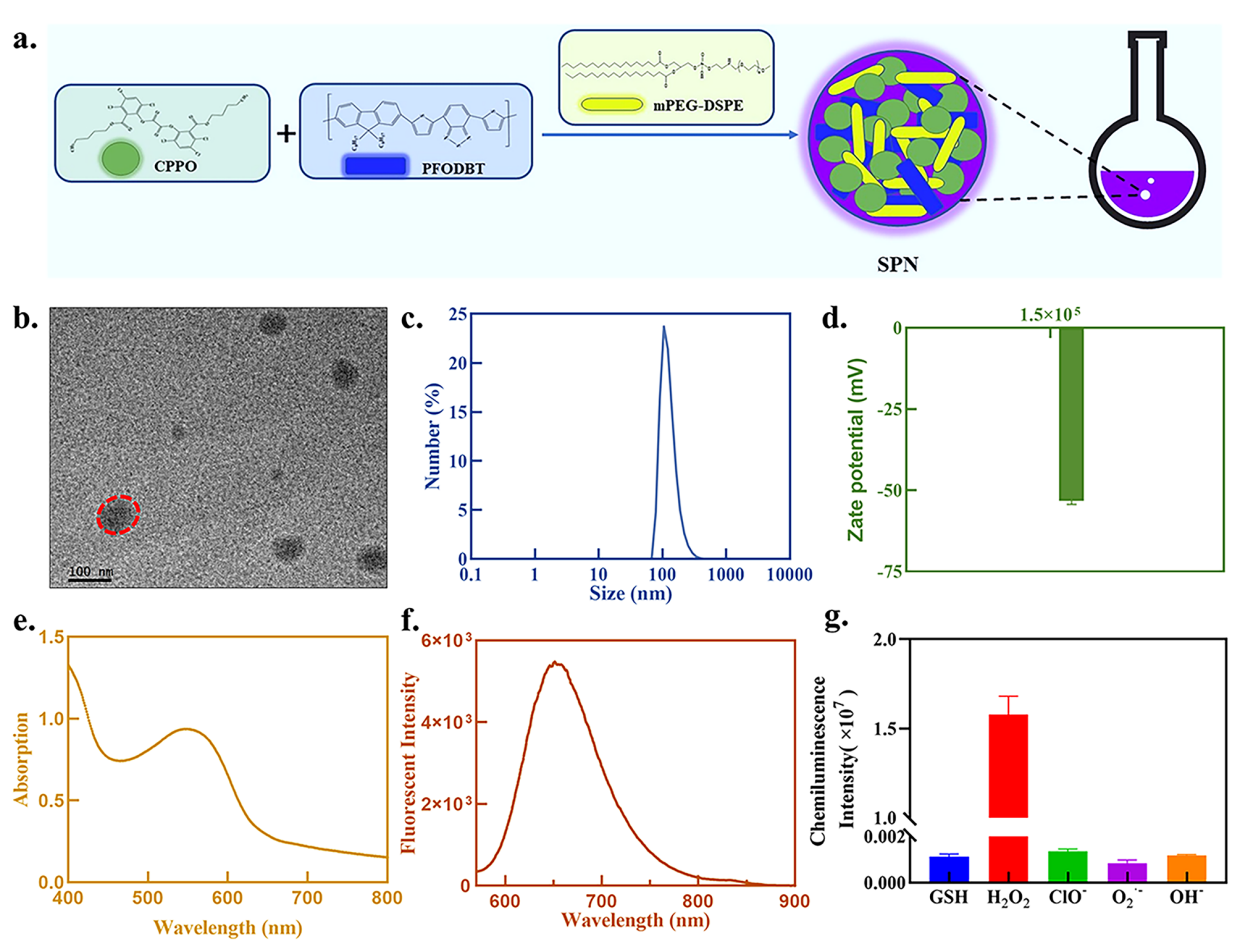
Syntheses and characteristics of the semiconducting polymer. nanoplatform. (**a**) Schematic of SPN preparation. (**b**) Transmission electron microscopy (TEM) image of the SPN. (**c**) Size of the SPN determined by DLS. (**d**) Zeta potential of the SPN in solution. (**e**) Absorbance spectra of the SPN. (**f**) Fluorescence spectra of the SPN. (**g**) Chemiluminescence selectivity of CPPO@PFO (CPPO/PFO = 40; 0.25 mg/mL PFO) incubated with various species, such as GSH, H_2_O_2_, ClO^−^, O_2_^·−^ and ·OH.

We designed and synthesized an integrated semiconducting polymer nanoplatform (SPN) with ROS-correlated CL to assess the severity of AP (Fig. 1). Our SPN CPPO@PFO was prepared according to the procedure illustrated in Fig. 2a. First, a typical nanoprecipitation reaction was used to synthesize nanoparticles by injection of tetrahydrofuran (THF) containing poly(2,7-(9,9-dioctylfluorene)-alt-4,7-bis(thiophen-2-yl)benzo-2,1,3-thiadiazole)(PFODBT) and bis(2,4,5-trichloro-6-(pentyloxycarbonyl)phenyl) oxalate (CPPO) in water under sonication. Moreover, 1,2-dimyristoyl-sn-glycero-3-phosphoethanolamine-N-[methoxy(poly(ethylene glycol))] (DSPE-PEG) was anchored to the surface of the polymer nanoparticles via hydrophobic interactions to endow them with aqueous solubility. Typically, CPPO@PFO exhibited a spherical morphology (Fig. 2b), and it had a size of 79–450 nm as determined by dynamic light scattering (DLS), among which the largest was 105 nm (Fig. 2c). In this nanoplatform, the CL substrate of CPPO was used as an energy donor and the near-infrared fluorescence emission of the semiconducting polymer (PFODBT) was used as an energy acceptor, enabling CRET from CPPO to PFODBT in the presence of H_2_O_2_ to emit near-infrared CL. The surface charge of CPPO@PFO was measured to be -52.10 mV (Fig. 2d). Moreover, the purple aqueous suspension of SPN exhibited strong absorbance in the ultraviolet (UV) region at approximately 553 nm and fluorescence spectra emission at approximately 651 nm (Fig. 2e and f). Notably, CPPO@PFO exhibited negligible reactions with various substances (GSH, H_2_O_2_, ClO^−^, O_2_^·−^ and ·OH each at a concentration of 1 µmol/mL) but was quite responsive to H_2_O_2_, indicating high selectivity for H_2_O_2_ (Fig. 2g).

### Optimization of the semiconducting polymer nanoparticles

**Fig. 3:**
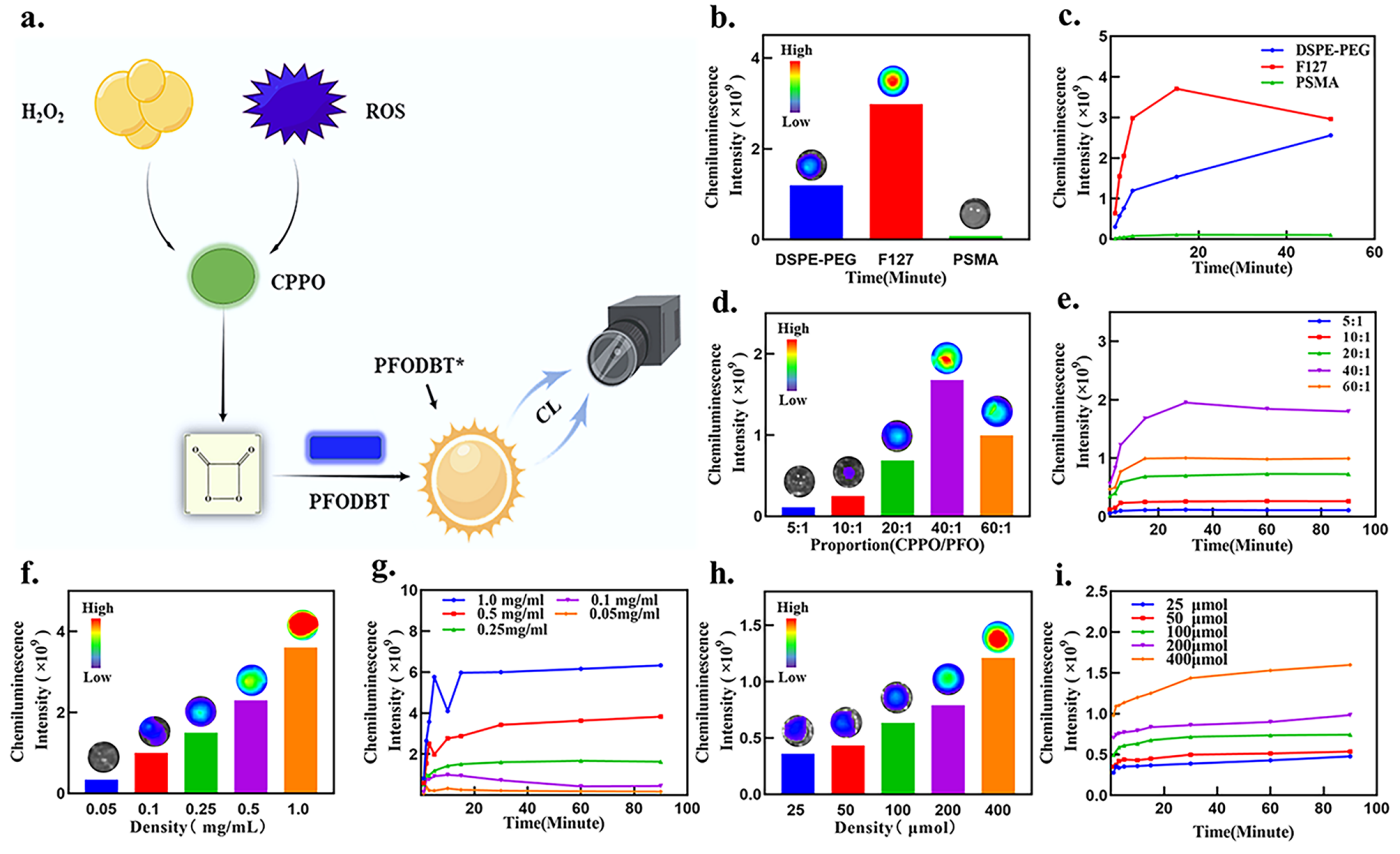
Optimization of the semiconducting polymer nanoparticles. (**a**) Schematic of the mechanism by which the ROS content was monitored by chemiluminescence. (**b-c**) Optimization of the surfactant to enhance the chemiluminescence intensity. (**d-e**) Optimization of the CPPO/PFO ratio to enhance the chemiluminescence intensity. (**f-g**) Optimization of the density of PFODBT to enhance the chemiluminescence intensity. (**h-i**) Sensitivity of the optimal concentration of SPN (CPPO/PFO = 40; 0.25 mg/mL PFO) to H_2_O_2_.

According to the CL mechanism of the SPN (Fig. 3a), we systematically optimized the SPN synthesis parameters to achieve good CL properties. First, three different surfactants (DSPE-PEG, F127, and PSMA) were chosen to assemble the nanoparticles. More precipitate was produced during the synthesis using PSMA, while no precipitate was found with the other surfactants. In the presence of H_2_O_2_, no CL signal was collected in the PSMA group, and the intensity of the signal was higher in the other groups (Fig. 3b). Moreover, the intensity of the signal in the F127 group increased quickly but began to decrease after approximately 15 min, while the luminescence signal in the DSPE-PEG group was more constant and continued to rise (Fig. 3c). Thus, we chose DSPE-PEG as the surfactant for the SPN. Subsequently, we began to verify the effect of different doping ratios of CPPO and PFODBT on the CL intensity. The PFODBT nanoparticles with different doping of CPPO exhibited increasing CL intensity as the doping ratio of CPPO/PFO increased (Fig. 3d), but 0.04 mg/µg CPPO/PFO resulted in the highest CL intensity, which stabilized with the extension of time (Fig. 3e). Considering that excessive CPPO would produce precipitate formation, 0.04 mg/µg CPPO/PFO doping was used for subsequent experiments. At the same ratio of CPPO/PFO and the same concentration of H_2_O_2_, the higher the PFO content was, the stronger the CL intensity (Fig. 3f). Although 0.5 µg/mL and 1 µg/mL PFODBT doping resulted in the highest CL intensity, such a large amount of PFODBT Induced the instability of the nanoparticles in solution, and some precipitation was observed during the synthesis process (Fig. 3g). Therefore, the optimal concentration of PFODBT was 0.25 µg/mL. Finally, according to the optimal solution obtained from the previous verification experiments, the sensitive SPN (CPPO/PFO = 40; 0.25 mg/mL PFO) reacts with different concentrations of H_2_O_2_. CL signals can be detected, among which the lowest concentration of H_2_O_2_ 25 µmol/mL was accurately detected (Fig. 3h).and with increasing time, the CL signal tended to stabilize (Fig. 3i).

### Safety of the semiconducting polymer nanoparticles in vitro and vivo

**Fig. 4:**
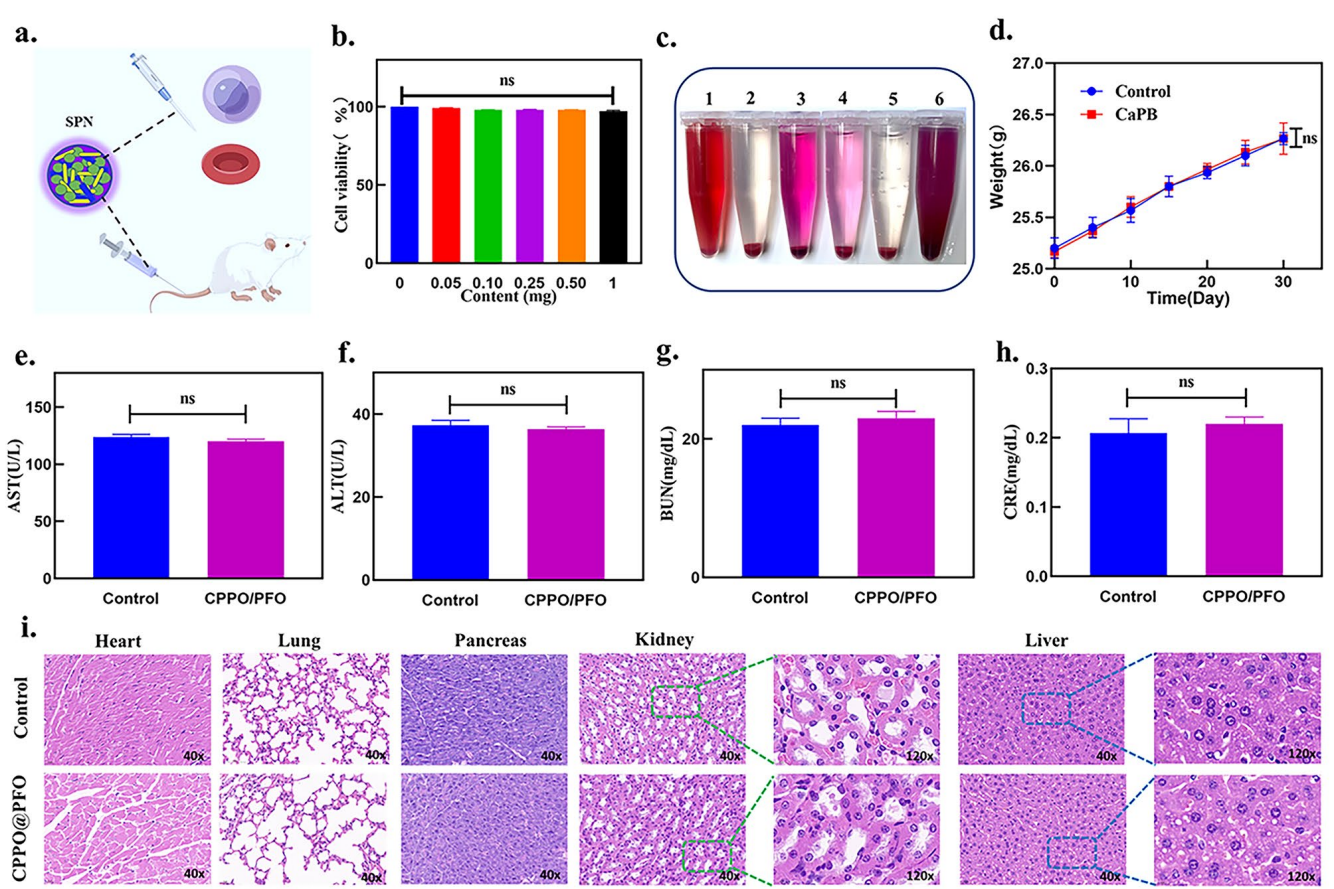
Safety of the SPN in vitro and in vivo. (**a**) SPN operation diagram. (**b**) Viability of AR42J cells after culture with different concentrations of SPN. (**c**) Photographs of RBC haemolysis after incubation with various solutions for approximately 4 h: [1] H_2_O [2], NaCl, [3] DMEM [4], RPMI-1640 [5], PBS, and [6] CPPO@PFO. (**d**) Changes in animal body weight recorded over 30 days (n = 3). (**e-h**) Serum analysis of AST, ALT, BUN and CRE in mice after SPN i.v. injection. (**i**) H&E-staining of the pancreas and other organs, including heart, liver, kidney, and lung, from mice 30 days after treatment with SPN. (Data represent means ± SDs, ns indicates no significant difference.)

Before evaluating the severity of AP, the in vitro and vivo biocompatibility of the SPN was examined (Fig. 4a). To verify the safety of the SPN in vitro, mouse-derived pancreatic exocrine cells (AR42J) were first treated with different concentrations of CPPO@PFO for 24 h and their viabilities were detected using a Cell Counting Kit-8 (CCK-8) cell counting reagent. It was determined that high concentrations of semiconductor polymer nanoparticles did not have obvious toxic effects on cells (Fig. 4b). Then different solutions (H_2_O, PBS, RPMI-1640, DMEM, NaCl, and CPPO@PFO) were added to erythrocytes from healthy humans for 4 h of incubation. Haemolysis was visually observed in only the H_2_O group and not in the other groups (Fig. 4c). Therefore, SPN had blood good compatibility.

Next, NaCl and SPN were injected into mice separately. Compared to the control group, there was no significant body weight loss after SPN treatment (Fig. 4d). Furthermore, all haematological parameters, including aspartate aminotransferase (AST), alanine aminotransferase (ALT), blood urea nitrogen (BUN) and creatinine (CRE), after SPN treatment were in the normal range (Fig. 4e-h). Additionally, H&E-stained images of the main organs (kidney, heart, liver, pancreas, and lung) were acquired to examine damage after SPN administration. Histological evaluation revealed that there were no significant morphological changes in the groups, especially in the pancreas, liver, and kidney, after i.v. injection of the SPN (Fig. 4i).

### Degree of inflammation and biodistribution of the semiconducting polymer nanoparticles in mice with acute pancreatitis

**Fig. 5:**
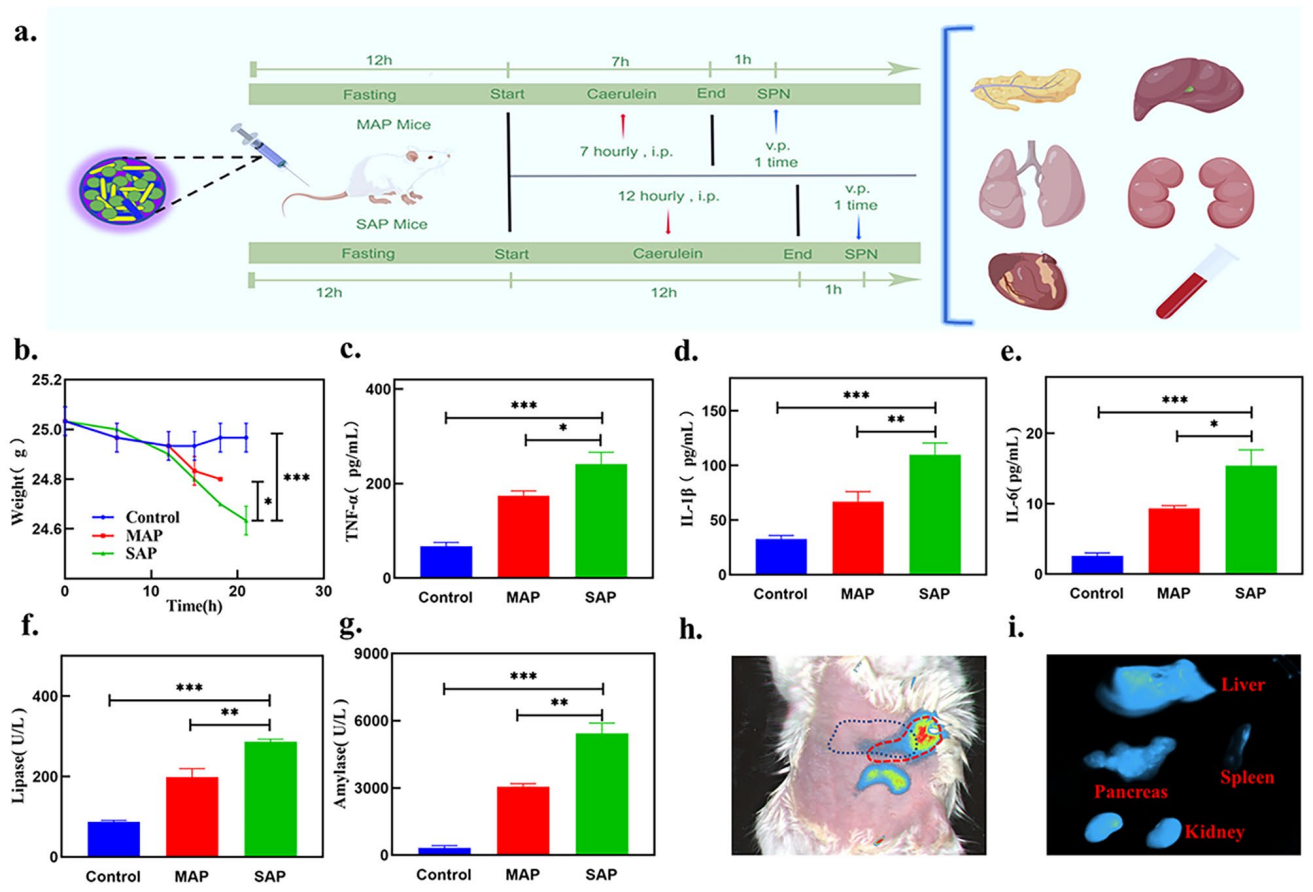
Degree of inflammation and biodistribution of the semiconducting polymer nanoparticles in mice with acute pancreatitis. (**a**) Experimental scheme by which the SPN assessed severity in AP mice. (**b**) Changes in body weight recorded over 21 h (n = 3). (**c-g**) Serum analysis of TNF-α, IL-1β, IL-6, lipase and amylase in mice of different groups after i.v. injection of SPN. (**h-i**) Fluorescence signal of the SPN in vitro and in vivo after i.v. injection to AP mice (blue dotted line: liver, red dotted line: pancreas). (Data represent means ± SDs, *p < 0.05, **p < 0.01, ***p < 0.001.)

Encouraged by the high-intensity CL in vitro and impressive biosafety in vitro and in vivo, we further investigated the ability of the SPN to assess the severity of acute pancreatitis in vivo. Using the caerulein-induced MAP and SAP mouse model, SPN was administered intravenously once (Fig. 5a). Compared to the control group, the mice in the MAP and SAP groups showed different degrees of weight loss (Fig. 5b). Then, the levels of inflammatory cytokines, including TNF-α, IL-1β and IL-6, were measured in the blood of the three groups of mice. The results showed that the levels of various proinflammatory factors in the control, MAP, and SAP groups were relatively elevated (Fig. 5c-e), and all the levels of proinflammatory factors increased with increasing severity in the mouse model. The serum levels of amylase and lipase in the MAP and SAP groups were higher than those in the control group (Fig. 5f, g). Following the injection of SPN through the tail vein, the fluorescence intensity increased in the liver, pancreas, and kidney of AP mice in vitro and in vivo, suggesting the targeted accumulation of the SPN (Fig. 5h, i).

### Ability of the semiconducting polymer nanoparticles to evaluate the severity of acute pancreatitis

**Fig. 6:**
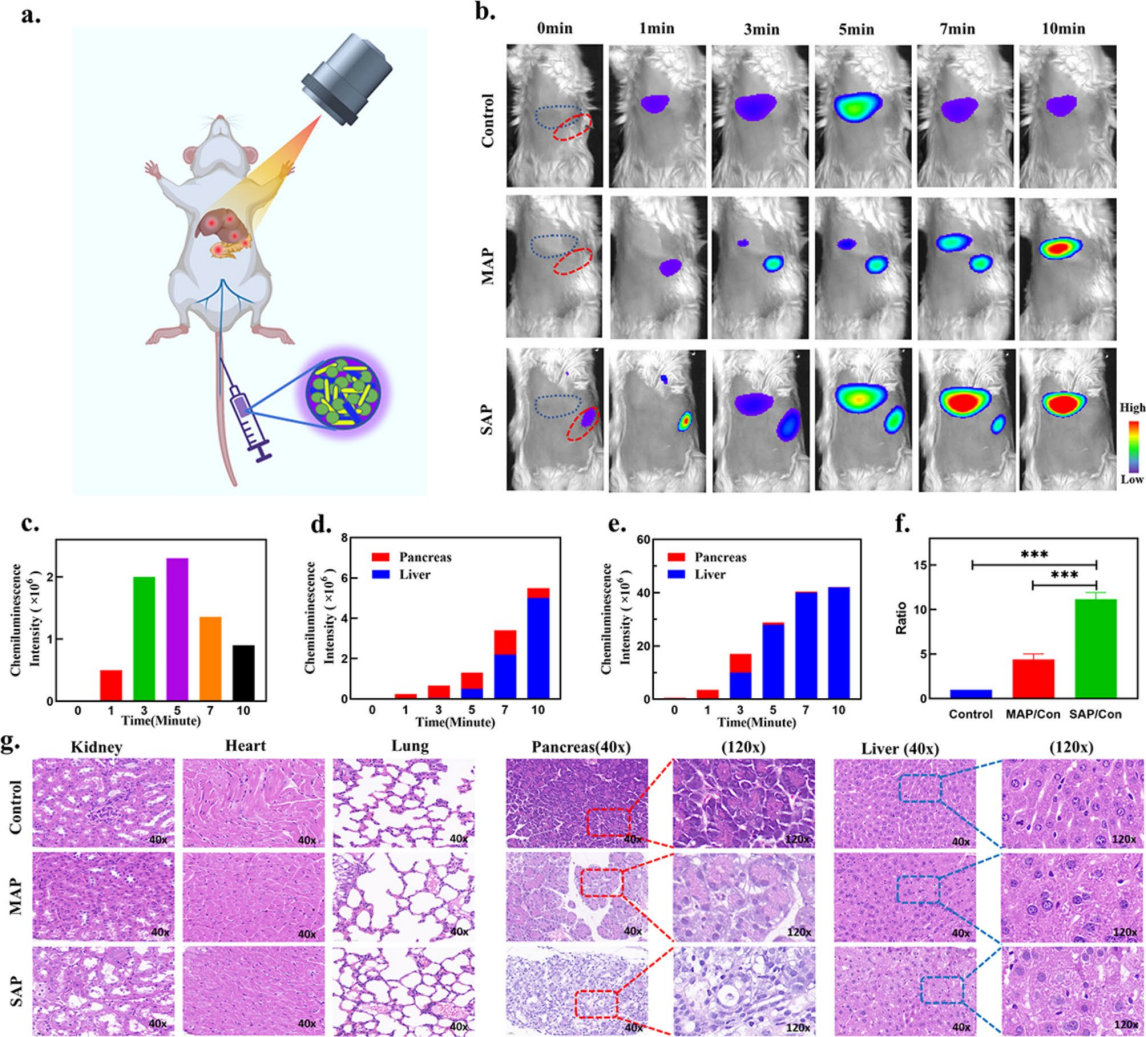
Ability of the semiconducting polymer nanoparticles to evaluate the severity of acute pancreatitis. (**a**) The reaction mechanism by which the ROS content was monitored by CL in vivo. (**b**) Chemiluminescence imaging of mice at different time points (blue dotted line: liver, red dotted line: pancreas, n = 3). (**c**) Corresponding quantification of the chemiluminescence intensity in the liver areas in the control group. (**d-e**) Corresponding quantification of the chemiluminescence intensity in the pancreas and liver areas in the MAP and SAP groups. (**f**) Ratio of the chemiluminescence intensity in the liver: MAP/control and SAP/control. (**g**) H&E-staining images of the pancreas and other organs, including the heart, liver, kidney and lung, in mice of the three groups. (Data represent means ± SDs, **p < 0.01, ***p < 0.001.)

Next, we explored the correlation between CL intensity and the severity of pancreatitis in vivo (Fig. 6a). From the CL images, one minute after i.v. injection of SPN, strong CL signals were observed in all three groups of mice. CL images were captured at the pancreatic site in both the MAP and SAP groups, and chemiluminescence was recorded at this site before the liver, compared to the control group. The CL of the pancreas appeared at approximately 5–10 min, and the CL signals in the three groups lasted approximately 20–30 min in the liver (Fig. 6b). In particular, no CL images were acquired from the pancreas of the control group of mice, while the liver signal peaked at 1 × 10^6^ for a shorter duration (Fig. 6c). The SAP group signal value in the pancreas of the mice was 6 × 10^6^, and the liver signal peaked at 1.5 × 10^7^ with persistent and stable CL (Fig. 6e). The intensity of the CL signal in the MAP group of mice was intermediate between the control group and the SAP group (Fig. 6d). With increased inflammation, the ratio of CL intensity also increased compared to that of the control group (Fig. 6f). Finally, histopathological changes in the pancreas and other organs in each group were analysed by H&E staining (Fig. 6g). Diffuse and localized oedema, swelling, and hardening of the pancreas were observed by the naked eye. After H&E-staining of the pancreas with different doses compared to the caerulein-induced AP model, we detected interstitial pancreatic oedema and neutrophil and lymphocyte infiltration in the interstitium and parenchyma, accompanied by variable degrees of haemorrhage and necrosis. Hepatocytes showed variable degrees of damage, ballooning degeneration, and even necrosis, along with inflammatory cell infiltration and fibrosis. Therefore, these results suggest that the intensity of the CL signals in different groups matched the extent of pancreatic inflammation.

## Discussion

In this study, we synthesized an integrated semiconducting polymer nanoplatform (SPN) with ROS-correlated CL for the early diagnosis and assessment of AP severity. The nanoplatform provided a correlation between CL intensity and the ROS content in solution, while rapidly collecting CL images at the site of inflammatory injury and to establish an excellent correlation between CL intensity and AP severity in vivo.

First according to assembly effect and efficiency we selected a stable and suitable surfactant, DSPE-PEG, which performed better than F127. Then, the formula of the SPN was optimized by increasing the ratio of CPPO/PFO and the concentration of PFO, which could enhance the CL energy transfer from CPPO to PFODBT and thereby improve the CL intensity, duration, and responsivity. Notably, a larger proportion of CPPO (CPPO/PFO = 60) resulted in a great decrease in CL, and a larger content of PFODBT doping (e.g., 0.5 and 1 mg/mL) resulted in the loss of stability, which is probably due to the concentration being too high. This led to a decrease in the SPN assembly rate and a decrease in CL intensity or instability. Excitingly, our nanoplatform has many excellent characteristics, such as tunable luminescence, high brightness, superior stability, and facile surface and intraparticle modifications, which enable the SPN to detect the biological parameters of ROS.

This SPN overcomes the extremely toxic reactions that characterize traditional CL systems and unwanted body clearance difficulty and has excellent biocompatibility in vitro and in vivo. There was no obvious harm to pancreatic acinar cells or red blood cells in vitro or major organs in vivo. The development of an integrated system for in vivo CL imaging and assessing the severity of AP is rare. We induced MAP and SAP in mice with different doses of caerulein. Based on the fluorescence image, the SPN had been transported to the main damaged organs through the blood. Subsequently, after injecting the optimal SPN by i.v. into different groups of AP mice, the CL signal was quickly collected, and the positions of the pancreas and liver were accurately imaged. Therefore, our nanoplatform overcomes the limitations of CT and MRI, which cannot diagnose AP in 72 h, improves the rate of diagnosis and prevents the onset of early AP [34]. We found that the CL intensity and lifespan in the pancreas and liver were significantly higher in the SAP group than in the control group, and those in the SAP group were higher than those in the MAP group. Thus, we can more accurately assess the level of pancreatitis inflammation through the signal ratio.

In our system, ROS play an important role. MAP and SAP mice have experienced varying degrees of oxidative stress, and the content of ROS in the pancreas and other organs are different in these groups [35, 36]. Thus, the content of ROS in vivo could be inferred based on the CL intensity, and then the severity of AP in mice could be evaluated. Moreover, by combining the inflammation indicators and the H&E-staining results among three groups, we can determine that the intensity of CL and the severity of AP are correlated. Thus, our nanoplatform avoids the requirements of excitation with an external light source and self-background interference and overcomes the drawback of AP diagnosis by serum amylase or lipase and imaging tests, which are unable to reflect the true severity of AP in time. Due to the complexity of AP diagnosis in the clinic, decisive quantitative methods to assess the severity of AP are lacking. However, after using more samples in the future, we believe that the results will be in line with expectations.

## Conclusion

In summary, we designed a SPN for CL imaging based on the correlation between the content of ROS and the severity of AP. The advantages of our nanoplatform can allow the main shortcomings of CL (low emission, flash-type emission, and a short wavelength) to be overcome and further promote CL imaging for in vivo utilization. Importantly, our nanoplatform was able to establish a good relationship between the CL imaging intensity and the content of ROS, and it could infer the degree of oxidative stress in vivo. Thus, this study reports on a facile strategy of using a SPN that enables CL imaging to assess AP severity. In the future, our nanoplatform may provide a new AP diagnosis method for use in clinical practice, as it achieves early AP diagnosis and predicts AP severity in patients by measuring CL intensity, and provides an advanced treatment option, thereby improving efficacy and prognosis.

## Data Availability

Data and materials are included in the manuscript.
